# Fetal heart rate monitoring of short term variation (STV): a methodological observational study

**DOI:** 10.1186/s12884-016-0845-8

**Published:** 2016-03-16

**Authors:** Stina Wretler, Malin Holzmann, Sophie Graner, Pelle Lindqvist, Susanne Falck, Lennart Nordström

**Affiliations:** Department of Women’s and Children’s Health, Karolinska Institutet, Karolinska University Hospital, 171 76 Stockholm, Sweden; Department of Medicine, Centre for Pharmacoepidemiology, Karolinska Institutet, Karolinska University Hospital, Stockholm, Sweden; Department of Clinical Science, Intervention and Technology (CLINTEC), Karolinska Institutet, Karolinska University Hospital, Stockholm, Sweden

**Keywords:** Cardiotocography, Computerized cardiotocography, Fetal hypoxia, Fetal heart rate, Fetal monitoring, Short term variation

## Abstract

**Background:**

Cardiotocography (CTG) has high sensitivity, but less specificity in detection of fetal hypoxia. There is need for adjunctive methods easy to apply during labor. Low fetal heart rate short term variation (STV) is predictive for hypoxia during the antenatal period. The objectives of our study were to methodologically evaluate monitoring of STV during labor and to compare two different monitors (Sonicaid™ and EDAN™) for antenatal use.

**Methods:**

A prospective observational study at the obstetric department, Karolinska University hospital, Stockholm (between September 2011 and April 2015). In 100 women of ≥ 36 weeks gestation, STV values were calculated during active labor. In a subset of 20 women we compared STV values between internal and external signal acquisition. Additionally we compared antenatal monitoring with two different monitors in another 20 women.

**Results:**

Median STV in 100 fetuses monitored with scalp electrode during labor (EDAN™) was 7.1 msec (range 1.3–25.9) with no difference between early (3–6 cm) and late (7–10 cm) labor (7.1 vs 6.8 msec; *p* = 0.80). STV calculated from scalp electrode signals were positively correlated with delta-STV (STV internal –external) (*R* = 0.70; *p* < 0.01). No significant differences were found between Sonicaid™ and EDAN™ in antenatal external monitoring of STV (median difference 0.9 msec, Spearman Rank Correlation Sonicaid vs delta-STV; *R* = 0.35; *p* = 0.14).

**Conclusions:**

Median intrapartum STV was 7.1 msec. Significant differences were found between internal and external signal acquisition, a finding that suggests further intrapartum studies to be analysed separately depending upon type of signal acquisition. Antenatal external monitoring with Sonicaid™ and EDAN™ indicates that the devices perform equally well in the identification of acidemic fetuses. Further studies are needed to assess the clinical value of intrapartum STV.

## Background

Detection of fetal hypoxia is of great importance in intrapartum surveillance. Cardiotocography (CTG) is often the method of choice in modern fetal monitoring [1–3]. CTG is a method with high sensitivity, i.e. most acidemic fetuses have CTG abnormalities, but less specificity [2, 4–6]. There is also poor inter- and intraobserver reproducibility in the interpretation of a CTG trace [7–13]. Computerized interpretation can be of help to eliminate the risk of human errors, and make the surveillance less user-dependent.

During the 1980ies Dawes and Redman [14–18] developed a method for antenatal computerized analysis in fetal heart rate monitoring (Sonicaid Fetalcare™, Huntleigh United Kingdom). A part of the Sonicaid™ system is the computerized calculation of short-term-variation (STV), i.e. beat-to-beat variation, a function which cannot be interpreted visually. Low STV (<3.0 msec) recorded in the antenatal period has been found to correlate to stillbirth and severe birth acidemia [15, 17–22]. However, so far the technique has only been applied in antenatal testing using an ultrasound device to monitor the fetal heart rate.

Recently a new CTG monitor (EDAN™ Instruments, China) was commercially launched, which can monitor STV both externally and with a scalp electrode. The scalp electrode uses the R-wave of the fetal ECG to calculate heart rate while external monitoring has a Doppler function which detects fetal cardiac structure movements as basis for calculation. The manufacturer highlights that STV analysis is not evaluated for intrapartum use. Analysis of STV in intrapartum monitoring is hitherto not properly studied [23, 24]. To the best of our knowledge STV has not been explored with signals derived from scalp electrode.

The main aim with the present paper was to study how we methodologically should derive signals for STV calculation during labor when trying to further explore the value of STV as an adjunctive method to CTG in detecting fetal hypoxia. Therefore we compared STV values calculated from signals simultaneously derived with external and internal monitoring. We also collected STV values during labor with scalp electrode and calculated their distribution. Additionally, we compared values simultaneously monitored externally in antenatal patients with two different monitors Sonicaid™ and EDAN™.

## Methods

This is a prospective observational study to evaluate STV as a method for intrapartum fetal monitoring. The study includes women at Karolinska University Hospital, Stockholm, Sweden between September 2011 and April 2015, a time period when 14.080 women delivered in our institution. All women were monitored according to national guidelines for CTG surveillance [25]. We choose to include only cases from first stage of labor to minimize signal loss, noise and other possible artifacts which might have complicated the methodological evaluation. All STV values were collected by two of the authors (SW, SF) and cases were included in the study when these colleagues were available. The STV values were concealed for staff involved in the clinical management of labor and delivery. The study was approved by the Regional Ethics committee in Stockholm (Dnr: 2014/2006-31/4). Participants gave oral consent to participate in the study, in accord with the ethical approval.

STV is the beat-to-beat variation in fetal heart rate. The CTG monitor calculates STV computerized by dividing every minute of the trace into 16 sections and the average pulse interval is calculated for every section. STV is the mean value of pulse interval differences between sections during one minute and the first value is displayed after 10 min recording. Thereafter new values are added and STV is updated continuously for up to 60 min. Detailed description of the algorithm is presented elsewhere [14–18]. Both Sonicaid™ and EDAN™ use this algorithm to estimate the STV. The Sonicaid™ monitor requires a signal loss of less than 30 % to allow calculation of STV. Corresponding figure for EDAN™ is less than 10 % [26]. EDAN™ is equipped for both external Doppler and scalp electrode monitoring including a twin function. Sonicaid™ is a CTG monitor developed for antenatal care with Doppler monitoring only. This monitor has no ability to use internal signal acquisition.

To evaluate STV methodologically the participants were grouped into three different sets.

STV values derived with scalp electrode during labor were collected in 100 women. Table 1 shows maternal characteristics, delivery and neonatal outcome data. The study population was chosen among women in labor who were eligible to be monitored with a fetal scalp electrode and the EDAN™ monitor available at the time. Inclusion criteria were term (≥37 week gestation), singleton pregnancy in cephalic presentation during first stage of labor.

**Table 1:** Background data of the study group with internal signal acquisition during labor for STV calculation

Population characteristics numbers and medians (range) *N* = 100	
Gestational age (days)	281 (261–296)
Maternal age (years)	31 (17–43)
Parity	
Nulliparous	62
Multiparous	38
Mode of delivery	
Spontaneous	61
Ventouse/forceps	21
Caesarean	18
Epidural	81
Oxytocin infusion	55
Apgar < 7 at 5 min	2
Admission to NICU	8
Birth weight (g)	3493 (2520–5030)

In 20 of these women we compared STV values derived with both external and internal monitoring. Monitoring with the twin function is more time consuming than the single function and the women included for this study had to be chosen when time and resources allowed it in a busy labor ward. With the twin function we recorded STV with both methods simultaneously from the same fetus.

The third part of the study is a comparison of external monitoring between the two different brands of monitors, Sonicaid™ and EDAN™. Simultaneous recordings were carried out in 20 women prior to labor. They all had singleton pregnancies with a gestational age of 36 weeks or more. Indications for monitoring were suspect but not confirmed ruptured membranes, breech presentation prior to external cephalic version, admission test before induction of labor and maternal diabetes.

### Statistics

Data are presented as numbers, medians/means and standard deviations, when appropriate, percentage and range. In comparison between continuous variables the Mann-Whitney *U*-test was used. Correlation between two parameters was calculated with Spearman Rank Correlation. Evaluation of agreement between different monitoring modes is displayed in Bland-Altman plots. Statistica™, version 12.0, (Statsoft Inc, USA) was used for the statistical analyses.

## Results

The median STV for all intrapartum cases (*N* = 100) was 7.1 msec (range 1.3–25.9). There was no difference in STV between early (cervix 3–6 cm) and late first stage of labor (7–10 cm) (median 7.1 vs. 6.8 msec; *p* = 0.80). Signal loss (EDAN™ monitor) was only available in 12 of the monitored cases (median 1.15 %; range 0–9.2 %).

**Fig. 1:**
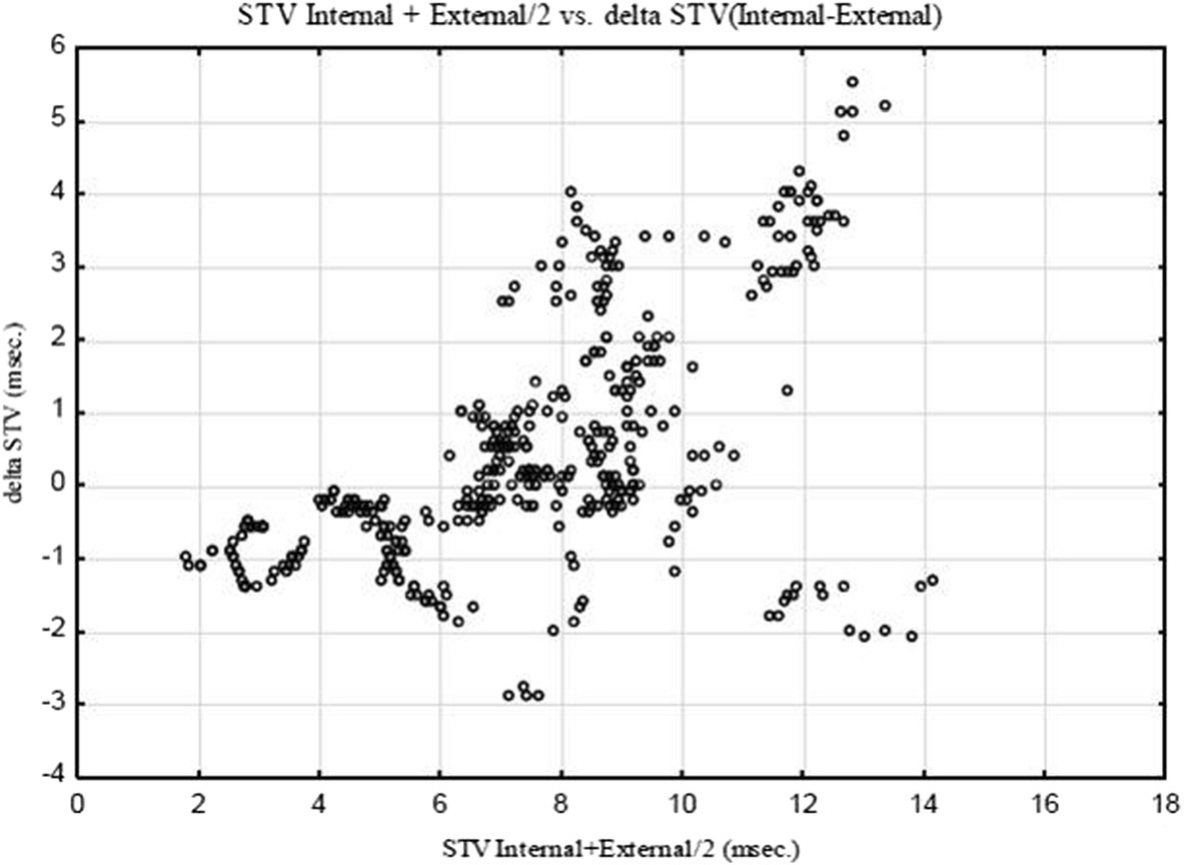
Internal vs. External monitoring of STV during labor. Bland-Altman plot of STV values (*n* = 463) calculated from signals derived with a scalp electrode and delta-STV (internal – external monitoring), using the twin function of EDAN™. Spearman Rank Correlation *R* = 0.70; *p* < 0.01

All 20 fetuses monitored with the twin function to enable simultaneous external and internal monitoring had at least 10 calculations of STV (in total 463 values). Median delta STV (internal-external) was 0.0 msec (range −2.9–5.5). However, in the lower distribution of STV (<8 msec) the scalp electrode derived values were found to be lower compared with the externally derived ones, while the opposite was found for higher values (≥8 msec). There was a significant positive correlation between STV from internal monitoring and delta STV (internal-external) (*R* = 0.70; *p* < 0.01) (Fig. 1). A similar correlation was found if only the first STV value per labor was included (*R* = 0.65; *p* < 0.01).

**Fig. 2:**
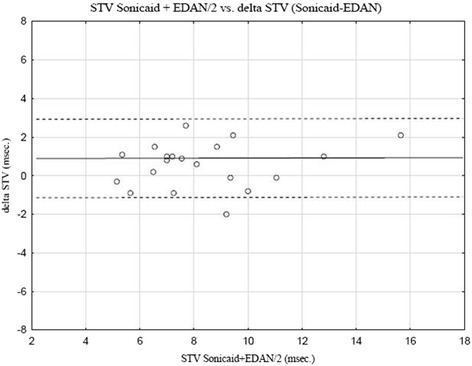
Antenatal STV monitoring with Sonicaid™ vs EDAN™. Bland-Altman plot with limits of agreement (±1.96 SD, dotted lines), of STV values (*n* = 20) simultaneously derived with external (ultrasound) signal acquisition with two different brands of CTG monitors, Sonicaid™ and EDAN™. Mean difference was 0.6 msec (SD ±1.17). Spearman Rank Correlation STV Sonicaid vs delta STV (Sonicaid-EDAN) *R* = 0.35; *p* = 0.14

Twenty pregnant women attending the outpatient clinic were simultaneously monitored externally with both Sonicaid™ and EDAN™. In these women the mean difference between STV Sonicaid and STV EDAN (delta-STV) was 0.6 msec (SD ±1.17) (median 0.9 msec) within a range of −2.0–2.6 msec. No significant correlation between STV Sonicaid™ and delta-STV was found (*R* = 0.35; *p* = 0.14) (Fig. 2). Signal loss was available in all cases monitored with Sonicaid™ (median 0.85 %; range 0.1–13.5 %). Only two of these had signal loss of more than 5 % (6.5 and 13.5 %, respectively).

## Discussion

We have presented single values of STV derived with a scalp electrode during labor with a median value of 7.1 msec (range 1.3-25.9). This is in agreement with data from a previous publication [23]. We found no difference in STV between early or late first stage of labor.

Due to the twin function we were able to do a simultaneous comparison of internal and external monitoring during labor. In this comparison we found that in the lower range of STV (<8 msec), the internal device calculated lower values than the external one, while higher STV values were found with scalp electrode in the higher range. We think that the difference detected is a true methodological error in the external device, as this method calculates STV from detected motions in fetal heart structures while internal monitoring is based on the fetal electrocardiogram, likely to be a more accurate method. Due to the rather strong correlation (*R* = 0.70) we do not think that extending number of observations would change our result. This finding is also supported by the clinical experience that when decreased baseline variability is found in a trace monitored externally application of a scalp electrode will usually display a trace with even lower baseline variability. This implies a need for different reference values for external and internal monitoring if STV is to be used in intrapartum fetal surveillance. Alternatively, STV should only be calculated during labor in cases with signal acquisition from a scalp electrode.

STV has so far been sparsely evaluated during labor [23, 24, 27]. These studies found no significant associations between STV and neonatal outcome. A likely explanation is the limited number of test cases, especially the few with birth acidemia, and how the algorithm has been applied. Our reference values in the present study are derived from a population with generally good neonatal outcome. These circumstances and the limited number of cases included imply that we cannot analyze the predictive properties of STV in the identification of acidemic fetuses during labor from the present data set.

In antenatal surveillance Sonicaid Fetal Care™ and EDAN™ showed good agreement between externally derived STV values. The largest difference between the monitors was about 2 msec. However these values were in the normal range of STV, while in the lower range the differences were smaller (Fig. 2). These minor differences might be due to precision of the Doppler equipment. Ideally we would have monitored a higher number of cases in the low range of STV, which is of clinical interest (<4 msec). However, these are rare findings in term and near term gestations, which was the target population in the present study.

A possible weakness in the comparison between the two machines for external monitoring is that differences in signal loss between the devices might have occurred. However, signal losses are less than required for the calculation algorithm as both monitors have accepted to calculate STV. Some degree of signal loss is also a reality in the clinical setting and not included in the evaluation of STV. As EDAN™ has strict criteria to allow STV calculations (signal loss <10 %) and we had to print traces to get this figure, instead of using the electronic central monitoring system where STV is displayed, we did record signal loss in only a limited number of cases. According to our findings of low proportion of single loss in all cases monitored with Sonicaid™ and in the subset with EDAN™ (and EDAN’s strict criteria), we do not think that signal loss could be a significant error in our evaluation. We believe that both brands of monitors can be used in antenatal fetal surveillance using the established cut-off values for intervention derived with the Sonicaid™ machine. The problem with poor inter- and intraobserver variation in CTG interpretation has been described in a large number of studies and computer analyses have been tested to reduce this problem [13, 28]. We believe that computer analysis could improve this drawback in the use of electronic fetal monitoring.

Our results suggest that intrapartum fetal heart rate monitoring with calculation of STV might need to have different reference values dependent upon type of signal acquisition, scalp electrode or external Doppler mode. To explore the predictive properties of STV during labor, reference values need to be derived from a larger cohort of fetal heart rate traces recorded with both scalp electrode and Doppler, and with a sufficient number of acidemic fetuses included. As more dynamic changes occur in fetal heart rate during labor compared with the antenatal period (primarily decelerations of reflex origin), the algorithm for STV calculation might need to be adjusted to exclude non-hypoxic changes. Such studies are in progress. For antenatal fetal heart rate monitoring Sonicaid™ and EDAN™ are likely to calculate STV equally good.

## Conclusion

Calculation of fetal heart rate short term variation, STV, differs between internal (scalp electrode) and external (Doppler) mode of signal acquisition. We found no differences in STV values between early and late labor. STV might have a clinical value in intrapartum fetal surveillance, but further studies of the correlation between STV and fetal acidemia are needed before it can be used in clinical practice.

## Abbreviations

CTG: cardiotocography
ECG: electrocardiogram
STV: short term variation
